# Role of Virulence Determinants in *Candida albicans*’ Resistance to Novel 2-bromo-2-chloro-2-(4-chlorophenylsulfonyl)-1-phenylethanone

**DOI:** 10.3390/jof3030032

**Published:** 2017-06-24

**Authors:** Monika Staniszewska, Małgorzata Bondaryk, Zbigniew Ochal

**Affiliations:** 1 National Institute of Public Health-National Institute of Hygiene, Chocimska 24, Warsaw 00-791, Poland; mbondaryk@pzh.gov.pl; 2 Faculty of Chemistry, Warsaw University of Technology, Noakowskiego 3, Warsaw 00-664, Poland; ochal@ch.pw.edu.pl

**Keywords:** *Candida albicans*, antifungal activity, Compound **4**, virulence factors, paradoxical growth

## Abstract

We investigated the role of *KEX2*, *SAP4-6*, *EFG1*, and *CPH1* in the virulence of *Candida* under a novel compound 2-bromo-2-chloro-2-(4-chlorophenylsulfonyl)-1-phenylethanone (Compound **4**). We examined whether the exposure of *C. albicans* cells to Compound **4**, non-cytotoxic to mammalian cells, reduces their adhesion to the human epithelium. We next assessed whether the exposure of *C. albicans* cells to Compound **4** modulates the anti-inflammatory response (IL-10) and induces human macrophages to respond to the *Candida* cells. There was a marked reduction in the growth of the *sap4Δsap5Δsap6Δ* mutant cells when incubated with Compound **4**. Under Compound **4** (minimal fungicidal concentration MFC = 0.5–16 µg/mL): (1) wild type strain SC5314 showed a resistant phenotype with down-regulation of the *KEX2* expression; (2) the following mutants of *C.* *albicans*: *sap4Δ*, *sap5Δ*, *sap6Δ*, and *cph1Δ* displayed decreased susceptibility with the paradoxical effect and up-regulation of the *KEX2* expression compared to SC5314; (3) the immune recognition of *C. albicans* by macrophages and (4) the stimulation of IL-10 were not blocked *ex vivo*. The effect of deleting *KEX2* in *C. albicans* had a minor impact on the direct activation of Compound **4**’s antifungal activity**.** The adhesion of *kex2Δ* is lower than that of the wild parental strain SC5314, and tends to decrease if grown in the presence of a sub-endpoint concentration of Compound **4**. Our results provide evidence that *SAP4–6* play a role as regulators of the anti-*Candida* resistance to Compound **4**. Compound **4** constitutes a suitable core to be further exploited for lead optimization to develop potent antimycotics.

## 1. Introduction

The emergence of antifungal resistance has driven the field to seek novel antifungal targets that could lead to the development of effective drugs with a new mode of action. Our recent findings [1,2] focused on the antifungal sulfones’ activity, dealing with multiple aspects of the virulence attributes and immune system evasion mechanisms employed by *C. albicans*. The studies [1,2] included diverse regulators of drug resistance in *C. albicans* such as: (1) lytic aspartic proteases (Saps), involved in the resistance of *C. albicans* to azoles (efflux pumps [3]); (2) subtilisin-type serine convertase Kex2 playing a role in the cell wall formation, interfering with Saps in the cell wall remodeling mechanisms, and responding to antifungal drugs [3,4,5]; (3) transcriptional regulators controlling hyphae formation, given that yeast-hyphae implicates morphological alterations (cell wall remodeling [5]); (4) development of biofilms overcoming environmental stress such as drug exposure and immune system attack (metabolic adaptation) [6]. These attributes include robust responses to local environmental stress exerted by sulfones upon *C. albicans*, the inactivation of which attenuates virulence. 

We previously showed [1] that the primary sulfones’ action mode relies on the modulation of programmed cell death. Sulfones specifically affected the fungal cell membrane without injury in mammalian cells [1], indicating that their less evolutionary conserved target is presented in fungi (focused on the yeast-specific regulators of programmed cell death). It was described [7] that apoptosis requires active proteins’ synthesis. Our most recent studies [1,8] indicated that sulfones are responsible for the induction of *KEX2,* which can be associated with apoptosis and related to increased levels of pro-apoptotic proteins playing a role in the DNA repair check point. The proteinase Kex2 is a regulatory endoproteinase located in the *trans*-Golgi network, with multiple regulatory functions in *Candida* cells. Based on our results [1], it was proven that Kex2 is crucial for *Candida* to cause tissue damage. With respect to sulfone activity, it is conceivable that when overexpressed, *KEX2* has an activity sufficient to sustain viability and hyphal growth of *Candida*. Moreover, we showed that the phenotype with attenuated *KEX2* (abolished *SAP* processing) exhibited weak adhesion regardless of the influence of 4-chloro-3-nitrophenyldifluoroiodomethyl sulfone [1,8]. Therefore, more studies are required to identify *KEX2*’s biological functions in the sulfone resistance mechanisms displayed by *C. albicans*.

Transcriptional factors *CPH1* and *EFG1* participate in different biological processes (fungal cell wall organization, pathogenesis, and regulation of biofilm formation). Since these regulators are unique for *C. albicans,* they have been considered as potential antifungal drug targets [9]. As it was shown that *EFG1* is also involved in antifungal resistance [10,11], and deletion of *EFG1* increased susceptibility of *C. albicans* to halogenated methyl sulfones, here we investigated whether it may produce a similar effect against 2-bromo-2-chloro-2-(4-chlorophenylsulfonyl)-1-phenylethanone (Compound **4**). In light of the above, we applied these approaches to study the effect of mutation in the genes of serine protease *KEX2* and aspartic proteases (*SAPs*) on the *Candida albicans* resistance mechanisms against Compound **4**. 

On the basis of our findings [2] and in continuation of our effort to develop an antifungal target of Compound **4**, we report herein on the in vitro susceptibility of the *C. albicans* wild type reference strain of SC5314 and mutant strains (*sap4Δsap5Δsap6Δ*, *efg1Δ*, *cph1Δ*, and *kex2Δ*) to Compound **4**. Additionally, screening against the *C. glabrata* wild type strain and mutants was included. We sought to determine whether the virulence factors (*KEX2*, *SAP4-6*, *CPH1*, and *EFG1*) could be associated with the paradoxical growth PG effect under sulfone influence. We tested the *Candida albicans* 90028 yeast cell growth inhibition under Compound **4** in time intervals. In addition, we also investigated the influence of Compound **4** on the *KEX2* transcription in the *C. albicans* sessile cells growing on the human epithelium layer (Caco-2). Under Compound **4** the mutants were tested for the *KEX2* role in the anti-sulfone response, including the adhesion to the epithelium and anti-inflammatory IL-10 stimulation. We examined whether the exposure of *C. albicans* cells to Compound **4** modulates the response of human macrophages against the *Candida* cells. 

## 2. Materials and Methods

### 2.1. Synthesis of 2-Bromo-2-Chloro-2-[(4-Chlorophenyl)Sulfonyl]-1-Phenylethanone (Compound **4**)

**Figure 1:**
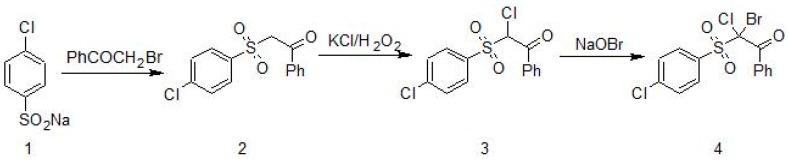
Synthesis of 2-bromo-2-chloro-2-(4-chlorophenylsulfonyl)-1-phenylethanone (**4**) [2].

As is shown in Figure 1, 2-bromo-2-chloro-2-(4-chlorophenylsulfonyl)-1-phenylethanone (Compound **4**) was synthesized starting from sodium 4-chlorobenzene sulfinate (**1**) which reacted with 2-bromo-1-phenylethanone to obtain 2-(4-chlorophenylsulfonyl)-1-phenylethanone; (**2**) was transformed into 2-chloro-2-(4-chlorophenylsulfonyl)-1-phenylethanone; (**3**) by the reaction with potassium halide in the presence of hydrogen peroxide and acetic acid. The final product; (**4**) was obtained by treatment of β-chloro (**3**) with sodium hypobromite [2].

### 2.2. Strains, Media, and Growth Conditions

The *Candida albicans* wild type reference strains, the mutants of *C. albicans* and *C. glabrata*, and the *Candida* clinical isolates used in the study are listed in Table 1. The decreased azole susceptibility *C. glabrata* strain was included in the study to further assess the antifungal activity of Compound **4**. Strains were stored on ceramic beads in a Microbank tube (Prolab Diagnostics, Richmond Hill, ON, Canada) at −70 °C. 

Prior to the respective examinations, routine cultures were conducted at 30 °C for 18 h in the Yeast extract-peptone-dextrose medium (YEPD) [19]. RPMI 1640 medium with l-glutamine without sodium bicarbonate (Sigma, Saint Louis, MO, USA) was buffered with 0.165 M morpholinepropanesulfonic acid (Sigma, Saint Louis, MO, USA) to a pH of 7. Stock solutions of Compound **4** and AmB (Sigma, Saint Louis, MO, USA) were prepared in dimethyl sulfoxide (DMSO, Sigma, Saint Louis, MO, USA) and RPMI, respectively, and stored at −20 °C until use.

### 2.3. Compound **4** Susceptibility Testing

Compound **4** activity against the planktonic cells of *Candida* was tested by the broth microdilution method M27-A3 [20]. Compound **4** was tested at concentrations that ranged from 0.25 to 16 μg/mL. As described in [2]: (1) compound test wells (PTW) were prepared with stock solution of Compound **4** (1600 µg/mL) dissolved in DMSO (66% in water), then the yeast cell suspension and Compound **4** (final dilution 1:100) were dispensed at a final volume of 250 µL/well into 96-well microplates; (2) growth control wells (GCW) were prepared with planktonic cells at a final density of 2.5 × 10^2^–2.5 × 10^3^ cfu/mL suspended in RPMI 1640 medium containing 0.66% DMSO (*v*/*v*); (3) sterility control wells (SCW) were made with Compound **4**, RPMI 1640 medium (Gibco^®^, Waltham, MA, USA), and sterile water replacing inoculum; (4) microtiter plates were incubated at 35 °C without agitation for 48 h. After incubation, the growth of the cells was measured by a microtiter plate reader Spark Control M10 (Tecan Group Ltd., Grödig, Austria). The endpoint was calculated as a 100% reduction in OD_405_ as compared to the growth in the control wells according to the formula: % of inhibition = 100—(OD_405_ CTW—OD_405_ SCW)/(OD_405_ GCW—OD_405_ SCW). Additionally, AmB (Sigma-Aldrich, St. Louis, MO, USA) at 0.5 μg/mL (MIC assessed visually, data not shown) against *C. albicans* SC5314 was tested. As described in [2], the minimal inhibitory concentrations (MICs) of the compound were read visually after 18 and 48 h. Paradoxical growth (PG), i.e., the reduced antifungal effect (<99.9% reduction of the starting inoculum) at concentrations of above clear endpoint (100% cell growth reduction) was evaluated. Tests were performed in three independent experiments.

### 2.4. Yeast Cell Growth Inhibition under Compound **4** in Time Intervals

We tested the *C. albicans* 90028 yeast cell growth inhibition under Compound **4** at the following concentrations: 32, 1, 0.0625, 0.0078, and 0.00195 µg/mL in time intervals [21]. AmB was used as a control against 90028. Furthermore, we used the untreated cells as a growth control. Briefly, the *Candida* cells were inoculated into the YEPD solid medium and incubated overnight at 35 °C. The final inoculum of 0.5 × 10^2^ to 2.5 × 10^3^ cfu/mL Sabouraud dextrose broth (Sigma-Aldrich, St. Louis, MO, USA) was prepared and placed into the microdilution plates that contained different Compound **4** concentrations, as described above. In addition, the growth control wells with DMSO were added. The visual results were further corroborated by using a spectrophotometer measuring the optical density at 405 nm during the period from 0 to 24 h of incubation. The latency period was defined as the time needed to reach the basal OD and start exponential growth [21]. All growth inhibition experiments were conducted in duplicates on different days and were presented as mean absorbance data.

### 2.5. Mononuclear Cell PBMCs. In Vitro Candida Stimulation

PBMCs were isolated from heparinized whole blood by gradient density centrifugation in Histopaque 1077 (Sigma-Aldrich, St. Louis, MO, USA) according to the manufacturer’s instructions. The quality of purification of PBMC was verified by staining the cell-containing slides with methylene blue and performing microscopic examination. The cells were counted to produce 5 × 10^5^ cells/mL. To differentiate PBMCs into the monocyte-derived macrophages in vitro, fresh PBMCs were distributed in 24-well plates, and were suspended in RPMI 1640 (Dutch modification, Sigma) supplemented with 2 mM l-glutamine, 1 mM sodium pyruvate, and 100 µg/mL gentamicin for the experiment stimulation [22]. The cells were seeded for one and half hours and then the cells were maintained in Dulbecco’s modified Eagle’s medium (DMEM) supplemented with 10% fetal bovine serum, 20 mM l-glutamine, and 100 µg/mL gentamicin (all from Gibco) at 37 °C and 5% CO_2_ (medium was changed every day). After 7 days of incubation at 37 °C, the macrophages were collected and seeded into coverslips and cultured for 30 min (in the latter conditions). Then, the macrophages were stimulated with the *Candida* cells (untreated and pretreated with Compound **4** for 18 h) at the ratio of 1:5 for 3 h at 37 °C in CO_2_. Experiments were performed in duplicate and repeated at least three times. The study was approved by the Ethics Committee of the National Institute of Public Health-National Institute of Hygiene (Opinion 5/2012, 28.06.2012 and Appendix 18.04.2013) and all donors signed an informed consent form.

### 2.6. Fluorescence Microscopy

Cells from different co-cultures on coverslips were stained with Acridine orange AO (Roche Diagnostics GmbH, Mannheim, Germany) working solution (0.01%) at low pH, contrasted by crystal violet and were used for examination under fluorescence microscopy (excitation/emission: at 520–650 nm). Briefly, the cells were washed with PBS and stained with AO for 5 min at room temperature. Then, after washing with PBS, contrast staining with crystal violet was performed for 3 min in the dark, and was followed by three times washing with PBS. Acridine Orange (AO) was used to identify engulfed apoptotic cells and visualized under fluorescent microscopy (Axioskop 40 Zeiss, Oberkochen, Germany). Experiments were performed in triplicate.

### 2.7. Cytokine Analysis

Fresh PBMC (derived as described above) at a density of 5 × 10^6^ cell/mL were pre-incubated with *C. albicans* (2 × 10^6^/mL) in RPMI 1640 (Dutch modification, Sigma) supplemented with 2 mM L-glutamine, 1 mM sodium pyruvate, and 100 µg/mL gentamicin in 96-well plates for 24 h at 37 °C in 5% CO_2_ [23]. The monocytes were stimulated with the heat-killed (at 60 °C for 1 h) or live *C. albicans* cells, and then pre-incubated or not with Compound **4** for 18 h. The supernatants and cells were then separated by centrifugation and the supernatant was stored at −20 °C until cytokine quantification was performed. The levels of human IL-10 were measured by the ELISA sandwich technique (PeproTech EC Ltd., London, UK) and the results were expressed in ng/mL, according to the manufacturer’s instructions. Plate readings were conducted at 450 nm in the Infinite M200 PRO NANOQuant (Tecan Group Ltd., Grödig, Austria). The experiments were repeated at least three times.

### 2.8. Assay of Candida Adherence to Human Line Caco-2 (ATCC HTB27, LGC, Poland). Inhibition of Adhesion: Microwell-Based Assay

The adherence of *C. albicans* and *C. glabrata* to the Caco-2 cell line (ATCC HTB-37TM) under Compound **4** at a concentration of 0.25 µg/mL was performed as described previously [24]. The adherence was calculated according to the formula: % adhesion = 100% × X adherent cells/X control cells [24]. Where: X control cells stand for the number of cfu/mL of PBS; X adherent cells stands for cells showing adhesion after treatment with Compound **4.** The adhesion assay was performed in triplicate and repeated at least twice.

### 2.9. Analysis of Gene Expression

Total RNA was extracted from cells after 18 h growth in YEPD at 30 °C as previously described [25]. Simultaneously, the overnight-grown cells in YEPD were washed with water and then 200 μL of the suspension was added to 1800 µL of RPMI (final density of 1−1.2 × 10^6^ cells/mL) and inoculated onto the Caco-2 monolayer. Incubation was conducted for 18 h at 37 °C until the RNA extraction.

For the cells pre-treated with Compound **4**, the blastoconidia (as described above) were suspended (1−1.2 × 10^6^ cells/mL) in the YEPD medium containing 0.25 µg/mL (final concentration) of Compound **4**. Then, after 2 h incubation with Compound **4**, the cells were washed with water, suspended in 2000 µL YEPD, and incubated for 18 h at 30 °C until the RNA extraction. Simultaneously, pre-incubated cells with 0.25 µg/mL Compound **4** were re-suspended in 2000 µL of RPMI and inoculated onto the Caco-2 monolayer for 18 h at 37 °C until RNA extraction. Prior to further examinations, *C. albicans* total RNA was stored at −20 °C. First-strand cDNA synthesis was performed using the Enhanced Avian HS RT-PCR kit (Sigma-Aldrich, St. Louis, MO, USA) according to the manufacturer’s instructions. The sequence of the primer set of *KEX2* was designed by using Primer3 (Primer-BLAST, NCBI, Table 2). The primer set of *ACT1* (Table 2) was used as described previously by Naglik et al. [26].

cDNA was quantified using the FastStart Essential DNA Green Master (Roche Diagnostics GmbH, Mannheim, Germany), according to the manufacturer’s instructions. Each reaction mixture (15 µL) contained FastStartTaq DNA polymerase, reaction buffer, dNTP mix, SYBR Green I dye, 2 µL (250 nM) of each primer, water PCR grade, and 60 ng of template cDNA [26]. For reliable normalization of the *KEX2* gene expression data, we used the housekeeping gene *ACT1* as the reference gene [26]. Real-time PCR reactions were performed as described previously by Naglik et al. [26]: at 95 °C for 15 min, followed by 45 cycles of 15 s at 94 °C and 1 min at 60 °C with the LightCycler 96 Instrument (Roche Diagnostics GmbH, Mannheim, Germany). The specificity of each primer pair was determined by the presence of a single melting temperature peak. The *C*_T_ values were provided from RT-PCR instrumentation and were imported into a Microsoft Excel 2010 spreadsheet. The relative quantification was calculated using an equation [27], where ΔC_T_ = Avg. *KEX2 C*_T_—Avg. *ACT1 C*_T_ and ΔΔ*C*_T_ = Δ*C*_T_—Δ*C*_T_ parental strain.

### 2.10. Statistical Analysis

All of the above experiments were repeated at least in triplicate on different days, with experimental values expressed as mean ± SD. The statistical differences between the control and the test values were determined by means of the Wilcoxon signed-rank matched-pair test. A *p* value of 0.05 was assumed as the threshold for differences.

## 3. Results

### 3.1. In Vitro Anti-Candida Activity of Compound **4**

For SC5314, the MIC value and clear endpoint (100% cell growth reduction) were not observed at the range of concentrations presented in Table 3. The screening of antifungal activity revealed phenotypes displaying the persistent growth at the supra-concentrations of the endpoint (Table 3). The incomplete growth inhibition observed at higher concentrations appeared to vary among the species and mutants and it depended on the deleted genes. Against most of the mutants tested, we observed the endpoint of Compound **4** at low concentrations of Compound **4** and its lower activity at higher concentrations (Table 3). The antifungal activity of Compound **4** (clear endpoint) was observed at 0.5 µg/mL against the null mutants *cph1Δ* and *sap6Δ* (PG phenotypes). We found that Compound **4** at 1 µg/mL inhibited the mutant cells completely (100% reduction for 5 out of 17 mutants tested). Thus a decrease in CFU count was observed mainly at 1 µg/mL followed by an increase in the number of CFU of the mutants at the higher concentration of 4 µg/mL (only 95–99% reduction, Table 3). The strain with *EFG1* reinsertion exhibited the growth inhibition of approximately 100% at 2 µg/mL (PG phenotype). The antifungal activity screening revealed the phenotypes showing persistent growth at the supra-concentrations of the endpoint (6 out of 17 strains tested, Table 3). The exceptions were the mutants as follows: *C. albicans sap4Δsap5Δsap6Δ*, *C. glabrata his3Δ trp1Δ kex2Δ::HIS3*, and *C. glabrata his3Δ trp1Δ::TRP1 kex2Δ::HIS3,* which revealed 100% reduction at 16 µg/mL. Precisely, this agent showed a clear endpoint against *C. glabrata his3Δ trp1Δ kex2Δ::HIS3* at 8 µg/mL and two-fold decreased activity (100% reduction at 16 µg/mL) against *C. glabrata his3Δ trp1Δ::TRP1 kex2Δ::HIS3* (Table 3). Although the latter *C. glabrata* mutants showed cell reduction at 16 µg/mL, the corresponding genes (*KEX2* and *TRP1*) reinsertion mutant was resistant at the whole range of concentrations tested. Thus the deletion of the *KEX2* gene in the cells of *C. glabrata* exerted sensitivity to Compound **4** (Table 3). On the contrary, all of the mutants of *C. albicans* attenuated in genes *KEX2* and/or *URA3* displayed the resistant phenotype under Compound **4** at the concentrations ranging from 0.5 to 16 µg/mL. Based on the antifungal activity assay, we further tested the influence of Compound **4** on: (1) *KEX2* expression; (2) adhesion; and (3) immune response.

### 3.2. Candida Cell Growth Inhibition under Compound **4**

**Figure 2:**
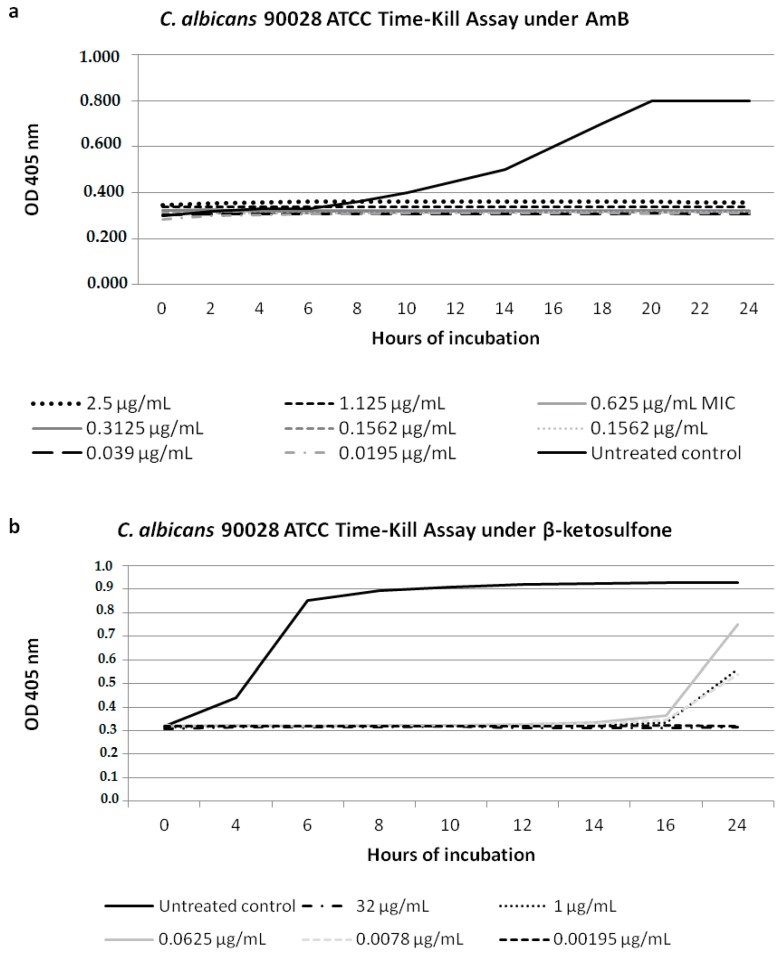
Characterization of growth of the *Candida* strains in the presence of Compound **4**. The suspensions were prepared with organisms in log phase growth and diluted in Sabouraud Dextrose Broth SDB medium to obtain a final inoculum of 2.5 × 10^2^−10^3^ cfu/mL (OD_405_ nm). The effect of the antifungal agent of AmB was used as a control growth inhibition assay during 24 h against the reference strain of 90028 (**a**); (**b**) Compound **4** showed a very good inhibitory effect against 90028. The antifungal nature of Compound **4** against 90028 was determined in the growth inhibition curve studies. Compound **4** was tested at the highest concentration of 32 µg/mL and at the lowest one of 0.00195 µg/mL.

We performed growth inhibition of the strain *C. albicans* 90028 under Compound **4** and AmB (Figure 2). For Compound **4** at the three concentrations tested (0.0078; 0.0625; 1 µg/mL) against *C. albicans* 90028, the latency period was 16 h, which was four times longer than that of the growth control (Figure 2b). A dramatic decrease in growth of 90028 was evident for Compound **4** at 32 µg/mL (Figure 2b). The cell growth inhibition for AmB at the concentrations ranging from 2.5 to 0.0195 µg/mL was shown (Figure 2a). We suggested that the difference in the latent period of 90028 depended on the temperature fluctuation going beyond our control during the incubation period.

### 3.3. Compound **4** Treatment Results in Inhibition of Adhesive Properties of Candida Strains

In the case of SC5314, the adhesion decreased 1.97-fold under Compound **4** at 0.25 µg/mL. The geometric mean adhesion level was 4-fold lower (*p* ≤ 0.05) for the mutant carrying the homozygous *KEX2* mutation (*kex2Δ*—CNA4) compared to the wild type strain SC5314. On the contrary, the heterozygous *URA3* mutation (*ura3Δ::URA3*—CNA1) decreased adhesion (1.3-fold) of the yeast cells to Caco-2, compared to SC5314 (Table 4). The CNA1 mutant untreated with Compound **4** displayed lightly reduced adhesion vs. SC5314. 

In contrast, the mutant reverted with one *KEX2* copy (*kex2Δ::KEX2—*CNA2) but carrying a homozygous *URA3* disruption (*ura3Δ—*CNA2) displayed significantly increased adhesion to Caco-2 (*p* ≤ 0.05). The mutants CNA1 and CNA3 (*kex2Δ/ura3Δ::URA3*) showed adhesion under these conditions, with the same level as the wild type strain SC5314 (*p* > 0.05, Table 4). The Compound **4-**treated-cells at 0.25 µg/mL showed significant changes in their adhesion properties, when compared with their non-treated counterparts (*p* ≤ 0.05, Table 4). Exceptions worth noting were as follows: the *C. albicans kex2Δ* reintegrated with one *URA3* copy (*kex2Δ/ura3Δ::URA3—*CNA3), and the cell attachment was not significantly inhibited (*p* > 0.05). The latter was similar for *C. glabrata kex2Δ* (strain 1006). The adhesion level of the mutants CNA1, CNA2, and CNA4 was inhibited as follows: 1.5-; 2.8-; 3-fold, respectively, at 0.25 µg/mL compared to their non-treated counterparts (Table 4). Additionally, the decreased adhesion was characteristic for *C. glabrata* 1008 (4.7-fold) and 1010 (2.9-fold). In the case of *C. albicans sapΔ*, the adhesion increased significantly (*p* ≤ 0.05) from 1.4- (*sap5Δ*) to 3.33-fold (*sap4Δsap5Δsap6Δ*) under 0.25 µg/mL. In contrast, in the *sap6Δ* mutant this feature was not significantly affected (*p* > 0.05). Pre-treating the cells with Compound **4** insignificantly affected the adhesion of *cph1Δ/efg1Δ*, compared with their non-treated counterparts (*p* > 0.05, Table 4). Interestingly, the remaining morphogenesis mutants displayed a statistically significant increase in the attachment to Caco-2 cells (*p* ≤ 0.05 compared to their untreated counterparts). In our adhesion assay all of the *C. albicans* cells displaying adherence to the epithelium were recovered on YEPD generating blastoconidial cells. Assessment of the number of viable cells (CFU) remaining after a 90-min pre-incubation with Compound **4** was carried out as described previously [24]. Ninety-minute incubation of the yeast suspension with Compound **4** did not decrease the viability of all of the tested strains (*p* > 0.05, data not shown). 

### 3.4. Interaction of C. albicans Cells with Macrophages and Fungal Morphology under Compound **4**

**Figure 3:**
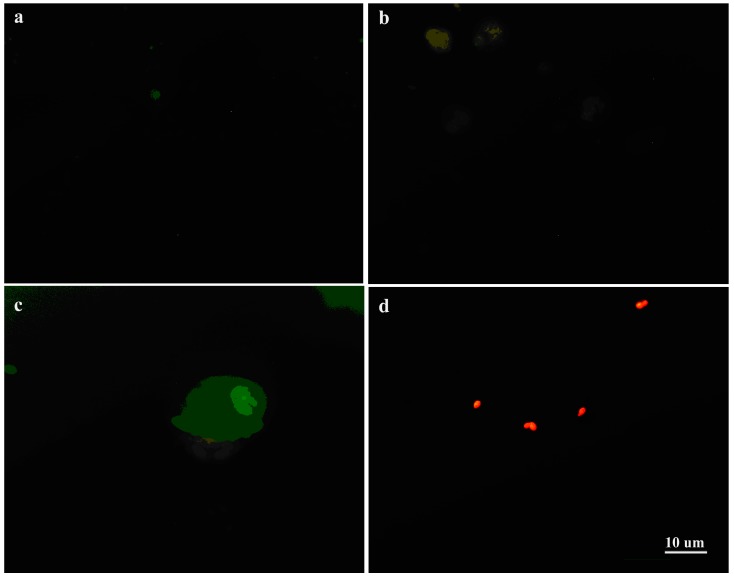
*Candida albicans—*Macrophage Interactions. *Candida albicans* cells were untreated or pre-treated with Compound **4**. (**a**) Control macrophage and *C. albicans* SC5314 cells (live) untreated with Compound **4** were seen in the macrophage’s vicinity; (**b**) Macrophages displaying signs of acidification (red fluorescence), suggesting that SC5314 cells treated with Compound **4** were ingested by them. Yeast cells showing acidification outside macrophages were observed (spotting red fluorescence); (**c**) Macrophages showed acidification (red fluorescence), suggesting that SC5314 cells untreated with Compound **4** were engulfed by it. Live yeast cells attached to macrophages were seen (green fluorescence); (**d**) SC5314 cells pre-treated with Compound **4** emitting red fluorescence.

Acridine orange fluorescent staining was applied to both the *Candida* cells treated and untreated with Compound **4** and incubated with macrophages (Figure 3). 

The *Candida* cells pretreated with Compound **4** were observed outside of the macrophages (Figure 3b,d). After 3 h of co-incubation of the *Candida* cells with the macrophages (Figure 3a,c), the cells were ingested or not by phagocytes. After pre-treatment with Compound **4**, the cells of the wild type SC5314 were observed outside of the phagocytes (Figure 3a,c). Moreover, we depicted the untreated cells of SC5314 not ingested by macrophages (Figure 3a,c), emitting green fluorescence outside of the macrophages. Noticeable, SC5314 pre-treated with Compound **4** showed cells outside of the macrophages (Figure 3d). Based on our results, we suggested that there were no differences in the interaction between macrophages and the *Candida* cells untreated and pre-treated with Compound **4**. Under microscopic examination, we found the *C. albicans* cells pre-treated with Compound **4** showing acidification, as detected by Acridine orange fluorescence (Figure 3d). Therefore, the cells exposed to Compound **4** prior to interactions with macrophages showed the plasma membrane and lysosomal membrane permeabilization causing the release of Acridine orange from the lysosomal lumen to the cytosol (Figure 3d). The argument is that, in the apoptotic cells, a proton pump increases the concentration of Acridine orange [28]. It appears that the Compound **4**-treated cells stained with Acridine orange showed a red-orange cytoplasm, and their nuclei stained yellow. Then, cell death was associated with the decrease in the pH of the cytoplasm (acidification), conceivably by fusion with lysosomes. Finally, this staining procedure identified and selected those cells at the apoptotic stage after the treatment with Compound **4**. 

### 3.5. Compound **4** Modulates the KEX2 mRNA Expression

**Figure 4:**
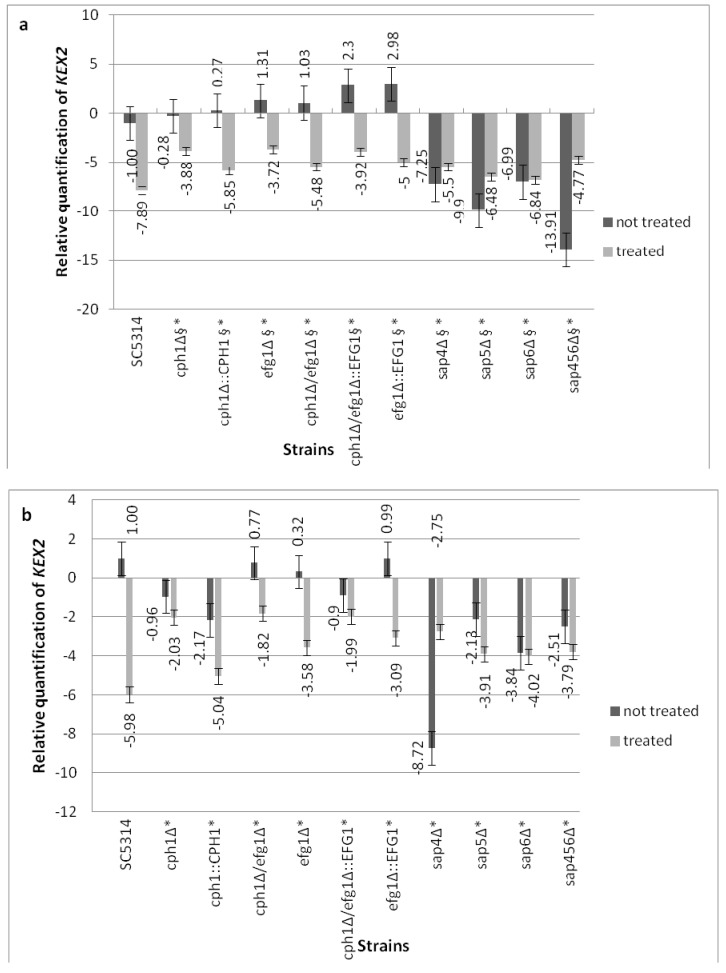
Expression level of *KEX2* normalized with the housekeeping gene *ACT1* for both the Compound **4**-treated (at 0.25 µg/mL) as well as untreated cells of *C. albicans*. (**a**) The transcript levels in the *C. albicans* cells growing in Yeast extract-peptone-dextrose growth YEPD medium; (**b**) The transcript levels in the *C. albicans* cells growing on Caco-2 cells; Legend: § means statistically significant differences between the untreated and treated cells (*p* < 0.05); * means statistically significant differences between SC5314 and mutants (both treated cells, *p* < 0.05).

The ability of *C. albicans* strains to express *KEX2* under Compound **4** was assessed with either the yeast cells pre-cultured in YEPD (Figure 4a) as well as in the cells growing on the Caco-2 monolayer (Figure 4b). To identify whether a certain class of genes (Figure 4a,b) was responsible for the *KEX2* response under Compound **4** influence, we compared the wild-type strain with either the morphogenetic mutants or the *sap4Δsap5Δsap6Δ* triple null mutant. Under Compound **4** influence, the mRNA level for *KEX2* was significantly affected in all of the mutants compared with the wild-type control cells. The decreased expression of *KEX2* was observed for all of the morphogenetic mutants (treated with Compound **4**) and significant differences (*p* < 0.05) appeared between the untreated and treated cells in both the tested media (Figure 4a,b). The *KEX2* ΔΔC_T_ raw data were monitored after 18 h growth of *C. albicans* (untreated and pre-treated with Compound **4**) in YEPD (Figure 4a). Firstly, the elevated *KEX2* expression by Compound **4** in *sap4Δsap5Δsap6Δ* during pre-culture in YEPD was noted; secondly, in the case of *sap6Δ*, almost comparable expression was observed between the treated and untreated cells. The *KEX2* expression analysis related to the *sap4Δsap5Δsap6Δ* treated cells grown on Caco-2 showed decreased levels compared with the untreated counterparts (except for *sap4*Δ showing three-fold increased expression vs. untreated cells, Figure 4b). Generally for Compound **4**, the transcript results of *KEX2* in all of the mutants tested were superior to the parental strain SC5314. Eighty percent viability of mammal epithelial cells was assessed for the exposure to Compound **4** at 0.25 µg/mL, as described in [15]. 

### 3.6. Interaction of Compound **4** with Interleukine 10

**Figure 5:**
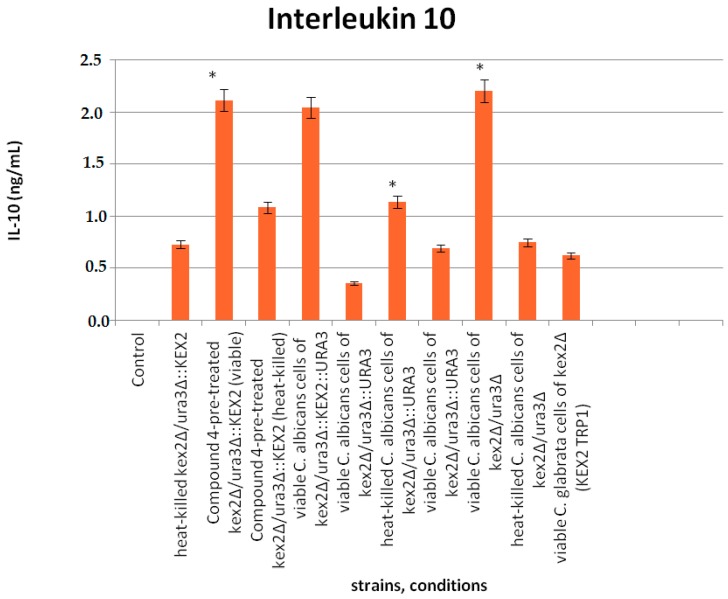
Production of IL-10 upon interaction of peripheral blood mononuclear cells PBMCs with the *Candida* mutant cells in the presence or absence of Compound **4** at 0.25 µg/mL. Legend: * stands for statistically significant differences between the viable mutants and their heat-killed counterparts in IL-10 induction. All data are presented as mean values ± standard deviations (SDs).

The experimental approach was conducted to evaluate whether Compound **4** affects the *Candida* mutant cells stimulating macrophages to produce IL-10 (Figure 5). The results indicated that IL-10 was significantly induced by the viable mutants (either untreated or pretreated with Compound **4**) compared with their heat-killed counterparts (*p* ≤ 0.05). No significant differences between the homozygous mutant *kex2Δ/ura3Δ* (CNA4) and heterozygous mutant reverted with one copy of *KEX2* (*ura3Δ/kex2Δ::KEX2*-strain CNA1) or another one complemented with one copy of both *KEX2* and *URA3* (*kex2Δ::KEX2/ura3Δ::URA3*—strain CNA1) either pretreated with Compound **4** and untreated were observed. Consistent with the results above, Compound **4** does not block IL-10 stimulation. The viable cells of the *kex2Δ* mutant complemented with one copy of *URA3* (*kex2Δ/ura3Δ::URA3—*strain CNA3) down-regulated IL-10 almost 6-fold compared with CNA1 and CNA2, respectively. The *C. glabrata kex2Δ* cells (heat-killed strain 1006) led to an insignificant IL-10 induction.

## 4. Discussion

We found that the following genes: *SAP4, SAP5, SAP6,* and *CPH1* are Compound **4** tolerance determinants, as planktonic cells of these mutants were characterized by a decreased Compound **4** sensitivity (PG effect noted at supra-endpoint concentrations) compared with SC5314. We showed that disruption of the morphogenic regulator *EFG1* does not enhance the sensitivity of *C. albicans* cells to Compound **4**. As reported previously [11], the sensitivity of *efg1Δ* to many classes of antimycotics remained unaffected. In our study, this mutant consistently showed a decreased sensitivity to Compound **4**, notwithstanding a possible involvement of a decreased content of ergosterol or any passive diffusion. The findings of the first report of Prasad et al. [11] suggested no noticeable difference in the membrane fluidity and passive diffusion in *efg1Δ* under the tested antimycotics. Following the previous findings [10] that Efg1 is involved in the regulation of the *ERG3* expression of the ergosterol biosynthesis pathway in *C. albicans,* we speculated that the action mode of Compound **4** might be different than targeting ergosterol or its metabolism. Moreover, in line with previous studies [11,22], we assumed that the unaltered (resistant) phenotype of the *C. albicans* cells under Compound **4** is not dependent on the drug efflux pump, which is affected by the mutation in *EFG1*. In view of this and of our above-presented findings, the phenomenon of being active in concert with the cell wall components remains a potential target for Compound **4** and its antifungal chemotherapy.

Considering the fact that the overall sensitivity of *sap4Δsap5Δsap6Δ* was restricted to Compound **4**, we speculated that these genes target the cell wall-associated genes, affecting its composition. Since we found that the adhesion of *cph1Δ* and *efg1Δ* was superior to that of the parental strain SC5314, we further suggested that the Efg1/Cph1 pathways might not be entirely responsible for adhesin release either under or without the influence of Compound **4**. The results obtained are in line with the findings of Carvalho et al. [29], which indicated another transcriptomic approach than *EFG1*/*CPH1,* acting under non-steroidal anti-inflammatory drugs. We suggested that Compound **4** interferes with the regulatory mechanisms in the *Candida* cells attenuated in the following genes: *SAP4, SAP5, and SAP6*, leading to a compensatory production of the virulence-related genes associated with the adherence, hyphal growth, and other proteases (*ALS3, ECE1, SAP1, SAP9,10*). Furthermore, Samaranayake et al. [30] reported that different *ALS* genes can complement each other.

We found that the overexpression of *KEX2* influences the susceptibility to Compound **4** in the s*ap4Δsap5Δsap6Δ* mutant or its single counterparts compared with the parental strain, depending on the growth medium and/or conditions. A possible explanation is that the upregulation of *KEX2* is required for processing of the virulence-related genes associated with the cell wall remodeling, adherence, and hyphal growth [18]. During the adaptive response to Compound **4** it is possible that *KEX2* provides modification to a maturation of the cell wall proteins (i.e., Sap5, Sap6, and Sap 9, 10), involved in the cell surface integrity. It is possible that the *KEX2*-dependent aspartic protease Sap2 can also be activated in *sap4Δ* under Compound **4**. Previous studies [19,28,31,32] showed an increased activity of Saps in the *Candida* strains resistant to antifungals. In addition, the Sap2 secretion is induced in the media containing proteins as the sole nitrogen source [33,34]. We are inclined to think that this enzyme can contribute to the *Candida* growth on the epithelial monolayer where the pH is reduced under Compound **4** (cell acidification).

Our study revealed a marked reduction in the activity of Compound **4** against the *C. albicans kex2Δ* mutant compared with the *C. glabrata kex2Δ* mutant (Table 3). The deletion of both *KEX2* and *URA3* directly activated the antifungal activity of Compound **4** (decreased adhesion, Table 4). However, no significant changes in the cell wall composition or immunogenicity of *C. albicans* were dependent on *URA3* [35]. *URA3* and *KEX2* are not an important aspect of the *Candida* biology stimulating PBMCs to produce anti-inflammatory IL-10 under the influence of Compound **4**. Herein, we did not test the direct impact of Compound **4** on the activity of monocytes/macrophages. Surprisingly, we found no differences in phagocytosis between the *Candida* cells treated with Compound **4** (physiologically inactive or dead) and those that were untreated.

Of further note, our findings were consistent with earlier reports [36,37] suggesting that PG represents a compensatory response to a range of cell wall insults. Since the wild type strain displayed a resistant phenotype under Compound **4**, the proper regulation of the cell wall integrity pathway is crucial to eliminate this effect. While the biological functions of Sap proteinases are still uncertain, we demonstrated that *SAP4*, *SAP5*, and *SAP6* can negatively regulate the maintenance of the cell surface integrity under Compound **4**. Our findings were consistent with earlier reports suggesting that this effect represents a generalized compensatory response to a range of cell wall insults, rather than specific responses to glucan synthesis.

## 5. Conclusions

We conclude that the molecular level changes of the signaling pathway can impact the paradoxical effect and overcome its negative effect on the treatment. We propose that the paradoxical effect may be due to a compensatory upregulation of the synthesis of the cell wall components in the *Candida* cells under the influence of Compound **4**. Compound **4** acting as *KEX2* inhibitor may prove useful for the treatment of *Candida* infections.

## Figures and Tables

**Table 1:** The strains used in this study.

Species	Strains	Parental	Genotype/Characteristic	Reference
*Candida albicans*	90028	None	Prototrophic wild-type strain	[12]
	SC5314	None	Prototrophic wild-type strain	[13]
	CAI4	SC5314	*ura3Δ::1imm434/ura3Δ::1imm434*	[14]
	*cph1*∆	CAI4	*ura3*Δ*::1imm434/ura3*Δ*::1imm434 cph1*Δ*::hisGΔ/cph1::hisG-URA3-hisG*	[15]
	*cph1*∆ (*CPH1*)	CAI4	*ura3*Δ*::1imm434/ura3*Δ*::1imm434 cph1*Δ*::hisG/cph1*Δ*::hisG (CPH1)*	[16]
	*efg1*∆	CAI4	*ura3*Δ*::1imm434/ura3*Δ*::1imm434 efg1*Δ*::hisG/efg1*Δ*::hisG-URA3-hisG*	[16]
	*cph1*∆*/efg1*∆	CAI4	*ura3*Δ*::1imm434/ura3*Δ*::1imm434Δ cph1::hisG/cph1*Δ*::hisG Δefg1::hisG/efg1*Δ*::hisG-URA3-hisG*	[16]
	*efg1*∆ (*EFG1*)	CAI4	*ura3*Δ*::1 imm434/ura3*Δ*::1 imm434Δ efg1::hisG/efg1*Δ*::hisG (EFG1)*	[16]
	*cph1*∆*/efg1*∆ (*EFG1*)	CAI4	*ura3*Δ*::1 imm434/ura3*Δ*::1 imm434 cph1*Δ*::hisG/cph1*Δ*::hisG efg1*Δ*::hisG/efg1*Δ*::hisG (EFG1)*	[16]
	*sap4*∆	SC5314	*sap4-1*Δ*::FRT/Δsap4-2::FRT*	[17]
	*sap5*∆	SC5314	*sap5-1*Δ*::FRT/Δsap5-2::FRT*	[17]
	*sap6*∆	SC5314	*sap6-1*Δ*::FRT/sap6-2* Δ*::FRT*	[17]
	*sap456*∆	SC5314	*sap4-1*Δ*::FRT/sap4-2*Δ*::FRT; Δsap5-1::FRT/sap5*Δ*-2::FRT; sap6-1*Δ*::FRT/sap6-2*Δ*::FRT*	[17]
	*kex2*∆CNA1	CAI4	*ura3*Δ*::imm434/ura3*Δ*::imm434KEX2kex2*Δ*::*his*GURA3*his*G*	[18]
	*kex2*∆CNA2	CAI4	*ura3*Δ*::imm434/ura3*Δ*::imm434KEX2kex2Δ::*his*G*	[18]
	*kex2*∆CNA3	CAI4	*ura3*Δ*::imm434/ura3*Δ*::imm434kex2*Δ*::hisGkex2*Δ*::hisGURA3hisG*	[18]
	*kex2*∆CNA4	CAI4	*ura3*Δ*::imm434/ura3*Δ*::imm434kex2*Δ*::hisGkex2*Δ*::hisG*	[18]
*Candida glabrata*	*kex2*∆1006	Cg*∆HT6*	*his3*Δ *rp1*Δt *kex2*Δ*::HIS3*	[5]
	*kex2*∆1008	1005	*his3* Δ*trp1*Δ *kex2*Δ*::HIS3* pKRT1(*KEX2 TRP1*)	[5]
	*kex2*∆1010	1005	*his3*Δ *trp1*Δ *kex2*Δ*::HIS3* pACT-14(*TRP1*)	[5]

**Table 2:** List of primers used in the study.

Primers	Sequence (5′-3′)
ACT1-1	GACAATTTCTCTTTCAGCACTAGTAGTGA
ACT1-2	GCTGGTAGAGACTTGACCAACCA
KEX2-1	TTTATATTGGGATATTTATTATCA
KEX2-2	TGGGATTTTAATAATAAAGGCAAA

**Table 3:** Antifungal activity (cell inhibition %; Means ± SD) of 2 bromo-2-chloro-2-(-4-chlorophenyl sulfonyl)-1-phenyloethanon (Compound **4**) against *Candida* strains after 48 h tested by using method M27-A3 [20].

*Candida* Spp.	µg/mL							
	16	8	4	2	1	0.5	0.5	0.5 ^1^
SC5314	99.69 ± 0.01	99.55 ± 0.03	95.01 ± 0.01	92.70 ± 0.21	93.14 ± 0.05	95.59 ± 0.01	93.50 ± 0.01	100
*Δsap4 ^3^*	97.97 ± 0.22	96.90 ± 0.02	95.50 ± 0.01	92.85 ± 0.02	100 ± 0.04 ^2^	99.90 ± 0.03	98.89 ± 0.02	100
*Δsap5 ^3^*	99.50 ± 0.00	99.31 ± 0.01	95.10 ± 0.20	40.05 ± 0.04	100 ± 0.03 ^2^	98.32 ± 0.10	97.00 ± 0.04	100
*Δsap6 ^3^*	99.80 ± 0.05	98.90 ± 0.01	99.42 ± 0.01	99.90 ± 0.01	96.95 ± 0.02	100 ± 0.01 ^2^	97.67 ± 0.01	100
*Δsap456 ^3^*	100 ± 0.02	98.70 ± 0.02	99.13 ± 0.01	99.85 ± 0.02	100 ± 0.01	97.89 ± 0.02	98.00 ± 0.04	100
Can16 ^3^	99.40 ± 0.03	99.42 ± 0.04	99.94 ± 0.05	100 ± 0.216	100 ± 0.01	100 ± 0.04 ^2^	99.00 ± 0.02	100
YLO323	99.71 ± 0.02	99.48 ± 0.03	95.67 ± 0.02	86.22 ± 0.02	92.04 ± 0.02	95.53 ± 0.22	95.67 ± 004	100
HLC52	99.63 ± 0.03	98.94 ± 0.04	99.48 ± 0.025	99.74 ± 0.06	99.77 ± 0.02	98.99 ± 0.04	98.90 ± 0.01	100
HLC54 ^3^	99.75 ± 0.01	99.16 ± 0.09	99.33 ± 0.08	99.83 ± 0.05	100 ± 0.06 ^2^	99.43 ± 0.09	99.00 ± 0.01	100
HLC74 ^3^	99.76 ± 0.01	98.93 ± 0.04	98.96 ± 0.04	100 ± 0.02	99.38 ± 0.01	98.85 ± 0.03	98.50 ± 0.03	100
HLC84	99.71 ± 0.02	99.31 ± 0.01	99.24 ± 0.02	98.86 ± 0.04	99.15 ± 0.02	99.16 ± 0.03	99.00 ± 0.01	100
CNA1	99.06 ± 0.00	99.04 ± 0.00	99.00 ± 0.01	98.98 ± 0.01	98.97 ± 0.01	98.96 ± 0.01	98.00 ± 0.02	100
CNA2	99.05 ± 0.00	99.00 ± 0.01	98.99 ± 0.01	98.98 ± 0.00	98.970 ± 0.00	98.95 ± 0.00	98.77 ± 0.03	100
CNA3	99.44 ± 0.00	99.19 ± 0.00	99.17 ± 0.00	98.98 ± 0.00	98.77 ± 0.01	96.50 ± 0.00	96.00 ± 0.01	100
CNA4	99.96 ± 0.01	99.48 ± 0.00	99.12 ± 0.01	99.11 ± 0.01	98.67 ± 0.01	95.64 ± 0.01	94.78 ± 0.01	100
1006	100 ± 0.00	100 ± 0.21 ^2^	99.47 ± 0.00	99.35 ± 0.01	99.27 ± 0.00	99.01 ± 0.01	99.00 ± 0.03	100
1008	99.08 ± 0.00	99.04 ± 0.01	98.99 ± 0.00	98.99 ± 0.01	98.97 ± 0.01	98.97 ± 0.00	97.90 ± 0.02	100
1010	100 ± 0.00 ^2^	99.27 ± 0.01	99.21 ± 0.00	99.16 ± 0.00	98.94 ± 0.01	98.94 ± 0.00	98.94 ± 0.03	100

^1^ Amphotericin B at a concentration of 0.5 µg/mL was used as a control (clear endpoint = cell growth inhibition at 100%); ^2^ stands for clear endpoint (cell growth inhibition % = 100); ^3^ stands for PG phenotype; Results were confirmed in three separate experiments on different days.

**Table 4:** The percentage of adhesion of *Candida* cells to the Caco-2 cell line after pre-treatment with 2-bromo-2-chloro-2-(4-chlorophenylsulfonyl)-1-phenylethanone (named Compound **4**). Adhesion data was calculated for cells grown on Sabouraud agar of a 24-well-plate. Adherence was expressed as a percentage of the total number of cells added (control cells non-treated). Data are expressed as the mean ± SD of three independent experiments. Values in bold indicate significantly affected adhesive properties compared to the non-treated counterparts (*p* ≤ 0.05).

Species	Strains	Genotypes	Cells Treated at 0.25 µg/mL	Non Treated Cells
*Candida albicans*	SC5314 (wt)	Prototrophic wild-type strain	**29.99 ± 0.34**	59.34 ± 0.06
	*sap4Δ*	*sap4-1*Δ*::FRT/Δsap4-2::FRT*	**76.67 ± 0.15**	46.32 ± 0.16
	*sap5Δ*	*sap5-1*Δ*::FRT/Δsap5-2::FRT*	**48.88 ± 0.17**	35.55 ± 0.21
	*sap6Δ*	*sap6-1*Δ*::FRT/sap6-2* Δ*::FRT*	25.29 ± 0.16	22.99 ± 0.04
	*sap4Δ/sap5Δ/sap6*	*sap4-1*Δ*::FRT/sap4-2*Δ*::FRT; Δsap5-1::FRT/sap5*Δ*-2::FRT; sap6-1*Δ*::FRT/sap6-2*Δ*::FRT*	**52.91 ± 0.33**	15.88 ± 0.03
	*cph1Δ*	*ura3*Δ*::1imm434/ura3*Δ*::1imm434 cph1*Δ*::hisG/cph1*Δ*::hisG*	**63.89 ± 0.25**	35.13 ± 0.24
	*cph1Δ::CPH1*	*ura3*Δ*::1imm434/ura3*Δ*::1imm434 cph1*Δ*::hisG/cph1*Δ*::hisG (CPH1)*	**30.56 ± 0.03**	12.82 ± 0.09
	*efg1Δ*	*ura3*Δ*::1imm434/ura3*Δ*::1imm434 efg1*Δ*::hisG/efg1*Δ*::hisG-URA3-hisG*	**46.40 ± 0.25**	29.26 ± 0.16
	*cph1Δ/efg1Δ*	*ura3*Δ*::1imm434/ura3*Δ*::1imm434Δ cph1::hisG/cph1*Δ*::hisG Δefg1::hisG/efg1*Δ*::hisG-URA3-hisG*	68.00 ± 0.16	72.21 ± 0.22
	*efg1Δ::EFG1*	*ura3*Δ*::1 imm434/ura3*Δ*::1 imm434Δ efg1::hisG/efg1*Δ*::hisG (EFG1)*	**48.33 ± 0.12**	8.34 ± 0.09
	*cph1Δ/efg1Δ::EFG1*	*ura3*Δ*::1 imm434/ura3*Δ*::1 imm434 cph1*Δ*::hisG/cph1*Δ*::hisG efg1*Δ*::hisG/efg1*Δ*::hisG (EFG1)*	**61.87 ± 0.38**	48.55 ± 0.078
	*kex2Δ* CNA1	*ura3*Δ*::imm434/ura3*Δ*::imm434KEX2kex2*Δ*::*his*GURA3*his*G*	**37.85 ± 0.04**	55.8 ± 0.18
	*kex2Δ* CNA2	*ura3*Δ*::imm434/ura3*Δ*::imm434KEX2kex2Δ::*his*G*	**22.49 ± 0.12**	62.45 ± 0.27
	*kex2Δ* CNA3	*ura3*Δ*::imm434/ura3*Δ*::imm434kex2*Δ*::hisGkex2*Δ*::hisGURA3hisG*	48.17 ± 0.41	52.33 ± 0.16
	*kex2Δ* CNA4	*ura3*Δ*::imm434/ura3*Δ*::imm434kex2*Δ*::hisGkex2*Δ*::hisG*	**7.33 ± 0.06**	22.20 ± 0.06
*Candida glabrata*	Cg *kex2Δ* 1006	*his3*Δ *rp1*Δt *kex2*Δ*::HIS3*	**45.13 ± 0.22**	56.99 ± 0.24
	Cg *kex2Δ* 1008	*his3*Δ*trp1*Δ *kex2*Δ*::HIS3* pKRT1 (*KEX2 TRP1*)	17.43 ± 0.08	82.15 ± 0.19
	Cg *kex2Δ* 1010	*his3*Δ *trp1*Δ *kex2*Δ*::HIS3* pACT-14 (*TRP1*)	**18.34 ± 0.07**	53.85 ± 0.28
